# Acknowledgement to Reviewers of *Bioengineering* in 2017

**DOI:** 10.3390/bioengineering5010005

**Published:** 2018-01-10

**Authors:** Bioengineering Editorial Office

**Affiliations:** MDPI AG, St. Alban-Anlage 66, 4052 Basel, Switzerland

Peer review is an essential part in the publication process, ensuring that *Bioengineering* maintains high quality standards for its published papers. In 2017, a total of 96 papers were published in the journal. Thanks to the cooperation of our reviewers, the median time to first decision was 20 days and the median time to publication was 52 days. The editors would like to express their sincere gratitude to the following reviewers for their time and dedication in 2017:
Acevedo, Rubén CarlosLee, Hae-HyoungAchinivu, EzinneLee, WonhyeAcosta-Vélez, GiovannyLeskinen, TimoAfonin, KirillLi, XueAfzal, Muhammad T.Li, ZhenyuAhn, Tae SeokLim, KhoonAiello, FrancescaLim, Seng HanAjao, Olumoye A.Lo, Kai-YinAkkus, AnnaLolla, DineshAl-Horani, RamiMadadi, HojjatAltmann, FriedrichMahimainathan, LeninArvidsson, Per I.Maia, FredericoAw, RochelleMajor, IanBai, OuMandenius, Carl FredrikBaldi, AlfonsoMarcos, MarcosBallesté, Ruben MasMarkoutsa, EleniBeatson, Richard E.Matsumoto, Ken'ichiroBenedetti, AnnaMazza, EdoardoBenz, HeatherMeiburger, Kristen M.Boehm, HeikeMejuto, Juan CarlosBommagani, ShbanbabuMelero, José AntonioBorghi, AlessandroMendonca, AntonioBrouzes, EricMeng, FanbenBrunella, ValentinaMiled, AmineBrunken, WilliamMilej, DanielCapablanca, LuciaMITANI, TomohikoCarosio, FedericoMobed-Miremadi, MaryamCarpenter, R. DanaMononen, MikaCasals-Terré, JasminaMorganti, PierfrancescoCasanella, RamonMorrison, Trudy G.Cerroni, BarbaraMuzykantov, VladimirChan, Hon FaiNaik, ShivangiChang, Young C.Nakas, James P.Charwat, VerenaNayar, SasiChaudhry, MuhammadNikkhah-Moshaie, RoozbehChaudhury, AnweshaObruca, StanislavChen, JuhongOehmen, AdrianChen, Yu-ChihOkafor, FlorenceChiessi, EsterOkesola, BabatundeCho, Dong-WooOkochi, MinaChristova-Bagdassarian, ValentinaOmasa, TakeshiChuburu, FrançoiseOstafin, Agnes E.Ciesielski, Peter N.Padmanabhan, JagannathClarke, AnthonyParisi, CristianCoburn, JeanninePascal, JorisCollins, David J.Pedersen, HenrikContino, AnnalindaPedersen, Thomas H.Creuzenet, CarolePeltokangas, MikkoDanilov, AlexanderPensabene, VirginiaDarling, Tom V.Picollet-D’hahan, NathalieDe Menezes, José CardosoPidaparti, RamanaDelville, Marie-HélènePittaccio, SimoneDessain, ScottPlaza, Gustavo R.Di Bella, ClaudiaPolacheck, WilliamDionisi, DavidePoole, KateEbara, M.Pradhan, ShantanuElgendi, MohamedPratt, StevenEstel, LionelPrigent, SylvainEsteve-Romero, JosepQian, BinzhiFarmer, Colleen G.Rahat, Michal A.Fenández-Tena, AnaRahman, ZiaurFerreira, Hugo A.Raina, MedhaFischlechner, MartinRainey, ThomasFonseca, Ana ClotildeRamirez-Vick, JaimeFortea-Verdejo, MartaReinhard, FriedemannFranze, KristianRipoll, LionelFriedli, ThomasRispoli, JosephFujiwara, KeiRittmann, SimonGao, YunxiangRoggendorf, HansGarcía Hidalgo, JavierRohrbaugh, JohnGarcia, AndresRomanowicz, PawelGargioli, CesareRotariu, CristianGargiulo, GaetanoRotermund, DavidGatenholm, PaulRuiz-García, AlejandroGerlach, JaredSaleh, Fatima A.Giannakoudakis, DimitriosSantos, LúciaGlover, PaulSau, SamareshGoldrick, StephenSaville, BradGomez-Ruiz, SantiagoSchulte, CarstenGonzalez, JuanSchwendeman, AnnaGoyanes, AlvaroSerrano, Dolores R.Guasto, JeffreyShah, PratikkumarGuerriero, JenniferShemfe, MobolajiGuo, XinxinShih, Tzu-ChingGupta, SwetaShimizu, KazuyukiHaggag, SherifSiegler, SorinHalldórsson, SkarphéðinnSimitzi, CharaHamon, MorganSimutis, RimvydasHayden, ERICSingh, YogeshHe, XiaomingSivan, UnnikrishnanHegedűs, CsabaSkoulou, Vassiliki K.Heim, FredericSmit, GerwinHendricks, MarkSteward, Robert L.Henning von Horsten, HansStrasser, RichardHerzberger, JanaSturm, GuidoHerzog, BastianSubramaniam, PrasadHeys, JeffreySun, ChenHickman, James J.Sur, ShantanuHimeno, HyoutaSvensson, KatrinHinderlich, StephanTabatabai, A. PashaHirabayashi, JunTahriri, MohammadrezaHissa, BarbaraTakemura, KenjiroHmaidouch, RimTan, Amy G.Y.Holnthoner, WolfgangTanaka, KenjiHong, QiutingTang, William C.Horvat, PredragTchuenbou-Magaia, FidelineHsiu, HsinTelegdi, JuditHsu, DanielTerrier, AlexandreHu, YangTeuschl, Andreas HerbertHuang, Yan Yan SheryThamaraparambil Jayaram, DhanyaHum, JasminThrivikraman, GreeshmaHyakutake, ManamiToya, YoshihiroIbarlucea, BergoiTsubaki, ShuntaroJacobsen, Elisabeth EgholmTuorto, FrancescaJeon, JessieUmezu, ShinjiroJha, Kshitij C.Vankemmelbeke, MireilleJoe, DanielVarache, MathieuJunne, StefanVasilateanu, AndreiKalogirou, OrestisVictor, BrunoKang, ChenVolpe, MaurizioKantak, ChaitanyaVon Stosch, MoritzKathuria, HimanshuWang, XinwenKehr, Nermin SedaWang, YangKevadiya, BhaveahWang, ZhijunKhisamutdinov, EmilWard, ChristopherKim, DongukWeir, ScottKinahan, David JWiedemann, PhilippKiparissides, CostasWinlove, PeterKitamura, ChiakiWitschey, Walter R.T.Kocot, JoannaWolfram, JoyKoh, Leng-DueiWu, YingKoller, MartinXing, ChangshengKontoravdi, CleoXing, XiaohuiKousiatza, CharoulaYang, GuangKremling, AndreasYang, ZixuKumi-Diaka, JamesYarema, KevinKundu, JoydipYi, Dong KeeKwon, Tae-yubYoon, SeongkyuLa Canna, GiovanniYuan, JunqiLangley, BenZafar, MuhammadLavvafi, HosseinZaucke, FrankLaycock, BronwynZhang, WenjunLe Tilly, VéroniqueZhao, HongyunLédé, JacquesZhu, WeiLee, Chul-HeeZhu, Xiangwei

